# The ultrathin limit of improper ferroelectricity

**DOI:** 10.1038/s41467-019-13474-x

**Published:** 2019-12-06

**Authors:** J. Nordlander, M. Campanini, M. D. Rossell, R. Erni, Q. N. Meier, A. Cano, N. A. Spaldin, M. Fiebig, M. Trassin

**Affiliations:** 1 Department of Materials, ETH Zurich, 8093 Zurich, Switzerland; 2 Electron Microscopy Center, Empa, 8600 Dübendorf, Switzerland; 3 Institut Néel, CNRS, 38042 Grenoble, France

**Keywords:** Condensed-matter physics, Ferroelectrics and multiferroics

## Abstract

The secondary nature of polarization in improper ferroelectrics promotes functional properties beyond those of conventional ferroelectrics. In technologically relevant ultrathin films, however, the improper ferroelectric behavior remains largely unexplored. Here, we probe the emergence of the coupled improper polarization and primary distortive order parameter in thin films of hexagonal YMnO_3_. Combining state-of-the-art in situ characterization techniques separately addressing the improper ferroelectric state and its distortive driving force, we reveal a pronounced thickness dependence of the improper polarization, which we show to originate from the strong modification of the primary order at epitaxial interfaces. Nanoscale confinement effects on the primary order parameter reduce the temperature of the phase transition, which we exploit to visualize its order-disorder character with atomic resolution. Our results advance the understanding of the evolution of improper ferroelectricity within the confinement of ultrathin films, which is essential for their successful implementation in nanoscale applications.

## Abstract (web summary)

Evolution of improper ferroelectricity within the confinement of ultrathin films is essential for their successful implementation in nanoscale applications. Here, the authors show thickness dependence of the improper polarization originating from the strong modification of the primary order at epitaxial interfaces.

## Introduction

In improper ferroelectrics, the polarization emerges through a coupling to another, leading order parameter, such as a non-ferroelectric crystal-lattice distortion, charge ordering, or certain spin arrangements^1,2^. The leading order can thus coerce the improper polarization into states that would be avoided in conventional ferroelectrics, allowing the emergence of exotic functional properties to complement those of conventional ferroelectrics, an issue of great interest for the development of next-generation oxide-based electronics^3–5^. In particular, improper ferroelectricity offers routes to achieving the coveted coexistence between electric and magnetic order^6–9^. Furthermore, unlike the polarization in thin proper ferroelectric layers, improper ferroelectricity is expected to exhibit a robustness against typical depolarizing-field effects, such as a critical thickness for spontaneous polarization^10–12^. All this makes improper ferroelectrics a class of materials of great interest, both for the fundamental understanding of complex ferroic order, as well as for their nanotechnological potential. Despite intense investigations of bulk improper ferroelectrics, however, it remains essentially unknown how their extraordinary properties transfer towards the technologically crucial limit of ultrathin films.

**Fig. 1:**
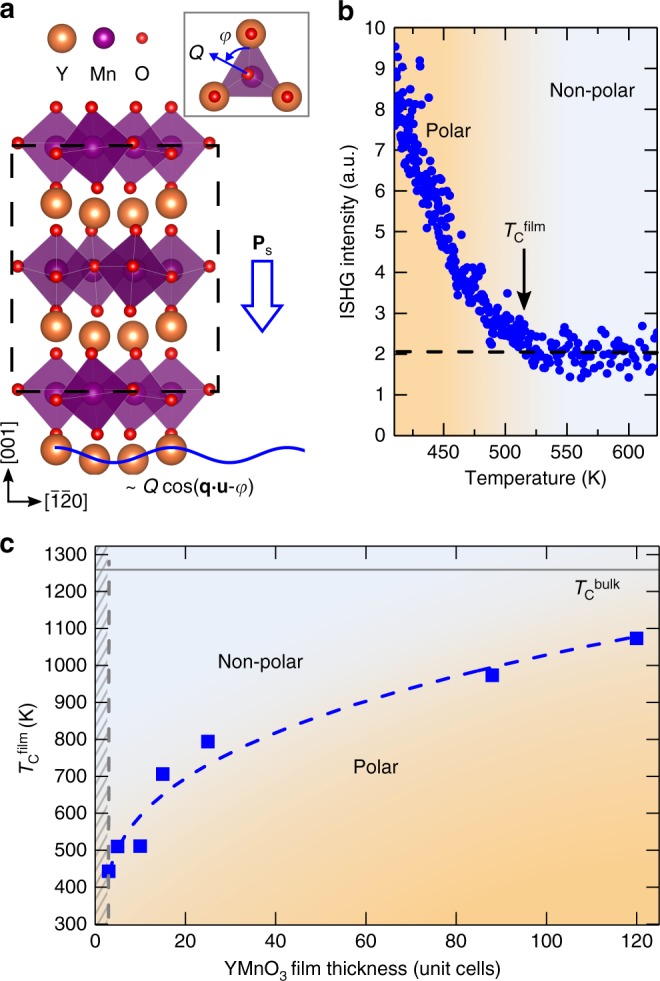
Evolution of improper polarization in YMnO_3_ thin films. **a** Schematic of the ferroelectric *P*6_3_*cm* YMnO_3_ crystal structure. The lattice trimerization **Q** is given by the tilt amplitude *Q* and azimuthal tilt angle *φ* of the MnO_5_ bipyramid (as defined in the inset viewed along [001]), and equivalently by the sinusoidal corrugation pattern of Y atoms. The dashed line outlines the YMnO_3_ unit cell. **b** Temperature dependence of the ISHG intensity from a 10-unit-cell YMnO_3_ film grown on YSZ upon cooling in the thin-film growth environment. The dashed line indicates the background level caused by surface contributions. The onset of **P**_s_ at the ferroelectric critical temperature *T*_C_^film^ (indicated by the arrow) breaks inversion symmetry, giving rise to an ISHG wave with an amplitude proportional to **P**_s_. **c** Ferroelectric transition temperatures for different YMnO_3_ film thicknesses. *T*_C_^film^ is extracted from ISHG measurements as in **a**. Films below three unit cells show no spontaneous polarization down to room temperature, indicated by the hatched gray area. Note that the thin-film crystalline quality could be maintained up to a thickness of 120 unit cells (<140 nm). Within this investigated range, *T*_C_^bulk^ = 1259 K (gray horizontal line) has not yet been reached. The dashed line, serving as a guide to the eye, highlights the thickness trend.

Among the different types of improper ferroelectricity, the hexagonal manganites are generally considered as a prototypical family of improper ferroelectrics with hexagonal YMnO_3_ being particularly well-studied. In this model material, the ferroelectric order is geometrically driven^13,14^. A leading-order structural trimerization of the lattice, described by a two-component order-parameter **Q**, parametrized by an amplitude *Q* and a phase *φ*^15^, lowers the YMnO_3_ symmetry from the centrosymmetric *P*6_3_/*mmc* to the non-centrosymmetric *P*6_3_*cm* space group (see Fig. 1a). As a secondary order parameter, the spontaneous polarization **P**_s_ emerges through its coupling to the leading-order parameter **Q**. The improper nature of **P**_s_ is key to the observed topologically protected ferroelectric domain-vortex pattern^16,17^, ferroelectric domain walls with tunable conductance^18–20^, and coexistence with magnetic order^2^.

However, the elevated ferroelectric transition temperature in bulk hexagonal manganites (*T*_C_^bulk^ = 1259 K for bulk YMnO_3_^21^) imposes major experimental requirements on the probing of the emerging improper polarization, and direct observation and comparison of the two order parameters at the phase transition point has remained elusive. In the ultrathin thickness regime, the challenge in characterizing the emergence of the improper polar state lies foremost in the difficulty in achieving epitaxial, single-crystal growth conditions from the first monolayer on appropriate substrates^22–24^. In earlier studies, analysis of the improper ferroelectric polarization was mainly limited to macroscopic, electrical measurements on films ≥100 nm^25,26^. There have been a few studies of the ultrathin regime, studying either the polarization^27,28^, or the lattice trimerization^29–31^. The microscopic interplay between the order parameters, however, and the consequences of their leading or secondary relation in the thin-film limit has to date not been explored. Hence, the consequences of finite thickness and the presence of hetero-interfaces on the improper polarization and its relation to the primary order-parameter are unclear.

Here, we probe the improper ferroelectric state in hexagonal YMnO_3_ thin films using a combination of two in situ probe techniques. Laser-optical second harmonic generation (SHG) with in situ monitoring of the thin films in the growth environment^32,33^ accesses the polarization, while high-resolution scanning transmission electron microscopy (STEM) with in situ heating of the thin-film samples independently measures the trimerization. Density-functional calculations based on the thin-film geometry help interpret the experimental results. We show that the substrate exerts a clamping of the YMnO_3_ unit cell at the thin-film interface which affects the build-up of the primary-order trimerization and leads to a strong thickness dependence of the improper polarization. By real-space visualization, we reveal an order–disorder type for the structural phase transition driving the emergence of polarization. Our results thus identify the dominant mechanisms steering the polarization in YMnO_3_ thin films and indicate the prospects of controlling the ultrathin regime of improper ferroelectricity in general by acting on its nonpolar primary order parameter rather than on the polarization itself.

## Results

### Emergence of improper polarization in the ultrathin regime

YMnO_3_ thin films are grown by pulsed laser deposition in the thickness range of 1–120 unit cells on (111)-oriented yttria-stabilized zirconia (YSZ) providing strain-relaxed epitaxial films (Supplementary Figs. 1 and 2). We achieve real-time film thickness control with sub-unit-cell precision through the use of in situ reflection high energy electron diffraction (RHEED) during the thin-film deposition. To detect the ferroelectric order in the samples, we use in situ SHG (ISHG) implemented in the growth chamber. This light-frequency-doubling process is sensitive to the breaking of inversion symmetry so that the amplitude of the ISHG light wave is proportional to **P**_s_^34,35^. ISHG thus has the advantage of being a nondestructive, contact-free probe of the pristine polar state (which distinguishes it from, e.g., scanning-probe techniques) over a large range of temperatures directly in the thin-film growth environment.

For each YMnO_3_ thin film, we extract the ferroelectric transition temperature (*T*_C_^film^) from the temperature-dependent onset of ISHG intensity measured during cooling from *T*_growth_ = 1070 K to room temperature (Fig. 1b, Supplementary Fig. 3 and Supplementary Note 1). The dependence of *T*_C_^film^ on the film thickness is shown in Fig. 1c. In all films, we observe ferroelectric phase transitions that are shifted to temperatures much lower than *T*_C_^bulk^. Even at 120 unit cells thickness, *T*_C_^film^ and *T*_C_^bulk^ differ by more than 100 K. *T*_C_^film^ decreases smoothly with YMnO_3_ film thickness and at room temperature, **P**_s_ reaches zero for the two-unit-cell film, thus establishing a threshold thickness for room-temperature ferroelectricity. To our knowledge, this is the lowest thickness for which an improper ferroelectric polarization in YMnO_3_ films has so far been stabilized.

These observations are reminiscent of depolarizing-field effects in conventional ferroelectric thin films^36^. In improper ferroelectrics, just as in conventional ferroelectrics, the spontaneous polarization **P**_s_ results in the build-up of a depolarizing field. However, the antagonism between **P**_s_ and the resulting depolarization field is shifted in favor of **P**_s_, because **P**_s_ is induced, and thus stabilized, by the primary order parameter, in this case the trimerization parameter **Q**. Since this leading-order parameter does not have a polarization, it is not strongly influenced by the electrostatics of the heterostructure. Therefore, while the depolarizing field is present, it is typically less effective in improper ferroelectrics, only supporting a possible small attenuation of **P**_s_ but not causing a critical thickness^11,12^. In the YMnO_3_ films we do observe a threshold thickness, however. This suggests two possible explanations. Either, the order parameters **Q** and **P**_s_ decouple in the thin-film limit and the polarization becomes proper and increasingly susceptible to the depolarizing field after all. Note that even for bulk crystals, a decoupling of **Q** and **P**_s_ was originally considered likely^13^. Alternatively, **Q** and **P**_s_ remain coupled and some other, yet unidentified mechanism is responsible for the suppression of **P**_s_ in the first two-unit cells of the YMnO_3_ thin films.

### Direct access to the lattice trimerization

**Fig. 2:**
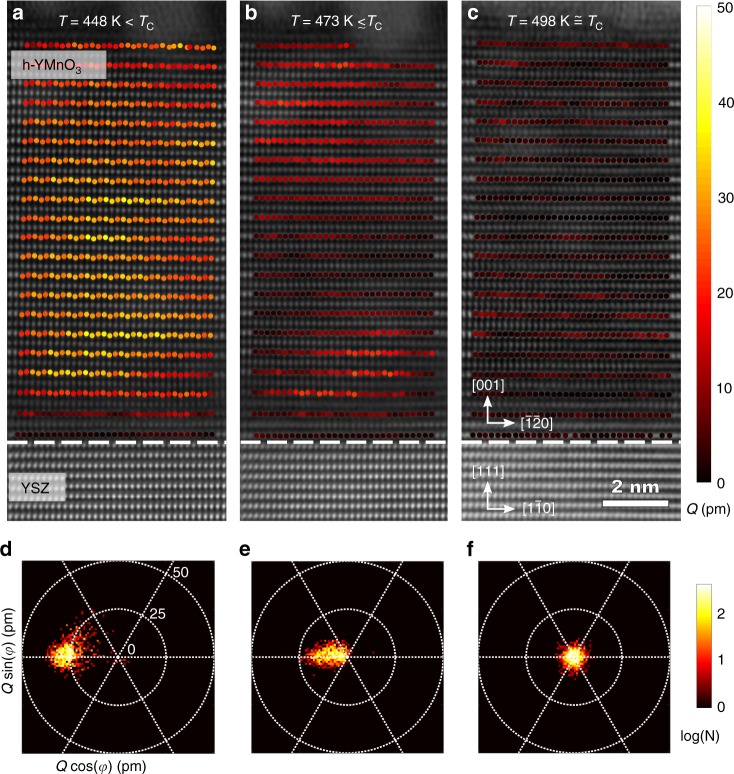
Real-space visualization of the lattice trimerization amplitude *Q* across the ferroelectric phase transition. **a**–**c** HAADF-STEM images of a 10-unit-cell YMnO_3_ thin film overlayed with the spatial distribution of the trimerization amplitude *Q* at the temperatures *T* = 448 K (**a**), 473 K (**b**), and 498 K (**c**), with *T*_Q_ = 498 K as phase transition temperature. **d**–**f** Histogram plots showing the number of occurences N of the *Q* and *φ* values (defined in Fig. 1a) for each temperature in **a**–**c**, respectively.

To clarify the relation between the polarization **P**_s_ and the crystal-lattice trimerization **Q** as the primary order parameter, independent access to the latter is required. We map this structural distortion in real-space using high-angle annular dark field (HAADF) STEM at atomic resolution, as a 10-unit-cell-thick sample is heated in situ. In the ferroelectric *P*6_3_*cm* phase of YMnO_3_, the trimerized lattice is recognized by its characteristic **Q**-related “up–up–down” displacement pattern of Y atoms parallel to [001] (see Fig. 1a), seen along the [100] zone axis (see Supplementary Note 2 and ref. 37). This corrugation of Y atoms constitutes a direct measurement of the leading-order-parameter **Q**^37^. The behavior of the primary order parameter with temperature can thus be directly visualized. Temperature-dependent fits of **Q**, shown in Fig. 2, reveal the gradual decrease of the lattice trimerization amplitude with increasing temperature (see also Supplementary Fig. 4). Comparing the temperature dependence of **Q** with the corresponding evolution of **P**_s_ from ISHG (see Fig. 1b) in the thin film, we see that the temperature *T*_Q_ at which **Q** reaches zero coincides with its ferroelectric transition temperature *T*_C_^film^, and hence the two order parameters emerge at a single transition point.

**Fig. 3:**
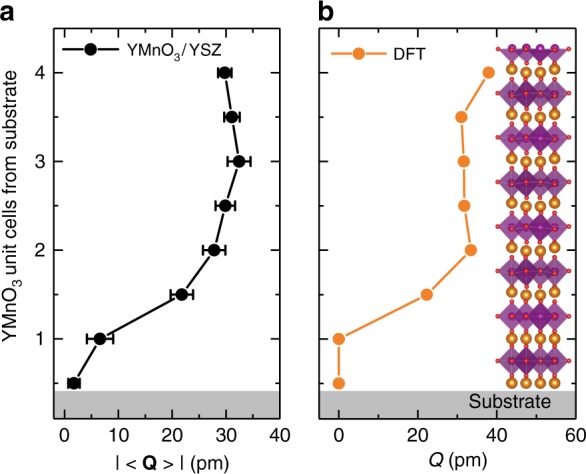
Influence of the substrate-film interface on the improper ferroelectricity. **a** Averaged displacement |**〈Q〉**| for each atomic row in the first four unit cells of the HAADF-STEM image in Fig. 2a. The value of |**〈Q〉**| is reduced at the substrate interface and saturates at the bulk displacement value after two unit cells. The error bars are given by the standard error of the mean. **b** Trimerization amplitude *Q* calculated by density-functional theory (DFT) for a four-unit-cell YMnO_3_ film keeping the bulk-lattice parameters and assuming a clamped (*Q* = 0) unit cell toward the substrate interface and a free top surface. The amplitude displays a progressive build up over the thickness of the film and saturates after two-unit cells. The inset in **b** shows a schematic of the corresponding crystal structure with atomic positions as calculated by DFT.

A similar experimental agreement is found between the observation of a threshold thickness for **P**_s_ (two-unit cells) and a suppression of **Q** close to the film-substrate interface. Figure 3a depicts the averaged displacement |**〈Q〉**| as a function of the distance from the substrate interface in Fig. 2a. Indeed, we see a complete quench of |**〈Q〉**| in the initial atomic layer, followed by a continuous increase over the first two-unit cells away from the interface, saturating at a bulk displacement of ~30 pm.

Based on these two observations, we conclude that we can relate the non-bulk-like value of *T*_C_^film^ and the threshold thickness of **P**_s_ (measured by ISHG) to the corresponding non-bulk-like value of *T*_Q_ and the thickness evolution of the primary order parameter **Q** (measured by STEM). In other words, we conclude that the improper relation between **P**_s_ and **Q** in the bulk^21^ is preserved down to the ultrathin limit, where the behavior of **P**_s_ in the thin films is directly guided by the primary structural order parameter **Q** and any mechanism acting on it. In particular, depolarizing-field effects causing the behavior of **P**_s_ to deviate from that of **Q**, are not observed^11^. This is further corroborated by the observation of similar multidomain structures in the films independent of the charge-screening efficiency of the substrate, see Supplementary Fig. 5.

### Impact of epitaxial interface on trimerization

Next, we use density-functional calculations to determine the mechanism for the thickness-dependent reduction of the transition temperature. Since our YMnO_3_ thin films are strain-free on YSZ substrates (as mentioned, they retain bulk-lattice parameters, see Supplementary Figs. 1 and 2), we exclude relaxation of epitaxial strain as the mechanism, and instead investigate the effect of mechanical clamping at the film-substrate interface on the lattice trimerization. We use density-functional theory (DFT) to calculate the magnitude of lattice distortion across a four-unit-cell film, modeling the interface with the substrate as a clamped, non-trimerized unit cell, with the termination at the surface of the YMnO_3_ film left free (see “Density-functional calculations” in Methods). We find, that such clamping results in a progressive suppression of the trimerization **Q** toward the interface with the substrate (Fig. 3b). This is consistent with the experimental data in Fig. 3a and explains the absence of **P**_s_ at room-temperature in the ultrathin regime, i.e., the presence of a threshold thickness.

Although suppression of the trimerization because of substrate clamping is expected to lead to a lowering of the transition temperature, such a clamping effect cannot fully account for the reduction of *T*_C_^film^ toward higher film thickness. We propose an additional thickness-related factor to explain this behavior, namely an increase in critical fluctuations of the order parameter as the system approaches the 2D limit. While such fluctuations also occur in conventional ferroelectrics, they are more likely to dominate in improper ferroelectrics, because the primary order parameter is not affected by the depolarizing field. Our preliminary calculations, considering critical fluctuations and using the Landau parameters determined from density-functional calculations^38^, are compatible with the observed scaling and the shift of *T*_C_^film^.

Hence, we see that the interface to the substrate and reduced dimension of the thin films have a profound impact on **Q**, and in turn on the improper spontaneous polarization through the coupling between **Q** and **P**_s_.

### Real-space observation of the structural phase transition

**Fig. 4:**
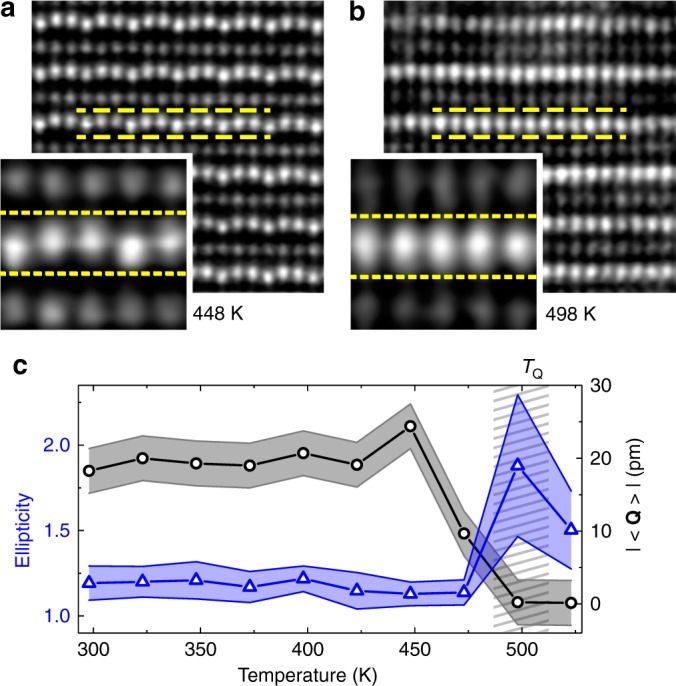
Real-space observation of the order–disorder-type phase transition. **a**, **b** The dashed yellow lines highlight the agreement between discrete displacement amplitudes in the ferroelectric phase (**a**) and the spread of the Y peaks in the paraelectric phase (**b**) in the 10-unit-cell YMnO_3_ thin film. An order–disorder character of the structural phase transition is revealed, where loss of long-range order (|**〈Q〉**|→ 0) is accompanied by the observation of elliptic Y atomic peaks due to superposition of a continuum of displacement states. **c** The average of **Q** (black circles) and average Y atomic peak ellipticity (blue triangles) from HAADF-STEM plotted as function of temperature for the same sample as in **a**, **b**. The increase of ellipticity arises directly as *T*_Q_ (indicated by the gray hatched area) is crossed. The error bars for |**〈Q〉**| correspond to the estimated experimental error in determining **Q** during the in situ heating experiment. The error bars of the ellipticity are given by the standard deviation.

Next, we exploit the lowering of transition temperature in the improper ferroelectric thin films to determine the nature of the improper ferroelectric phase transition in the YMnO_3_ system. We use the thickness dependence of the transition temperature to push *T*_C_^film^ to experimentally accessible temperatures, where we can monitor the paraelectric state at *T* > *T*_C_^film^ directly, using in situ STEM (Fig. 4a, b). We find that the atomic positions of the Y atoms do not lose their displacement amplitude in the non-polar *P*6_3_/*mmc* phase above *T*_C_^film^. Instead, we observe a spread of intensity of the Y peaks along the *c*-axis direction. The resulting ellipticity of the atomic peaks arises precisely at *T*_C_^film^ (Fig. 4c) and corresponds to a superposition of atomic states covering the same range of displacement amplitudes as observed in the ordered phase at lower temperatures. Evidently, the displacement of Y atoms is locally preserved in the paraelectric phase but the long-range order is lost. This is a signature of a specific type of order–disorder (here, Z_6_ → U(1)) transition: the amplitude *Q* is uniformly nonzero on either side of the phase transition whereas *φ* exhibits long-range order, taking on one of the six allowed trimerization angles (*n*∙ *π*/3; *n* = 0, 1, …, 5) below the transition temperature and disorder, with a random distribution of *φ* between 0 and 2*π*, above it (Supplementary Fig. 6). Such behavior has also been recently reported from neutron scattering experiments for bulk YMnO_3_^39^, and here we now observe it directly and in real space.

## Discussion

In conclusion, we have provided insight into key features of the evolution of improper ferroelectricity in ultrathin YMnO_3_ by achieving independent access to the leading and the dependent order parameters. On the microscopic level, we have shown how interface clamping and film-thickness effects act directly on the leading order parameter, the lattice trimerization expressed by **Q**. We furthermore identify these effects as the dominant mechanisms defining the nanoscale ferroelectric properties in YMnO_3_, in terms of its ferroelectric transition temperature and threshold thickness. We have exploited the reduced transition temperature in the thin films to directly visualize the phase transition with atomic resolution and establish that it is of a particular order–disorder type.

The results presented here point to the criteria setting the lower thickness limits for insertion of geometrically driven improper ferroelectric layers in nanotechnological heterostructures. Rather than the depolarizing field providing the main challenge to achieving polar thin films, as in proper ferroelectrics, improper ferroelectricity in ultrathin films is determined by the thin-film-specific behavior of its driving, non-polar order parameter. The generality of our findings is corroborated by our ongoing experiments on different substrates and using other compounds of the hexagonal-manganite series.

Our results thus indicate that the improper nature of a ferroelectric state can be used for steering polarization in nanotechnologically relevant ultrathin films through a depolarization-free primary order parameter. For example, the leading-order structural distortions may be tuned by choosing a differently sized rare-earth or transition-metal ion than Y or by insertion of spacer or capping layers^25^ with different mechanical boundary conditions, independent of their respective charge-screening properties. These approaches available to control improper ferroelectricity in thin films may provide additional degrees of freedom complementing the possibilities available to conventional ferroelectrics.

## Methods

### Thin-film growth and structural characterization

The YMnO_3_ thin films were grown on YSZ(111) substrates by pulsed laser deposition (PLD) using a KrF excimer laser at 248 nm, an energy fluence of 0.5–0.7 J/cm^2^ and a repetition rate of 8 Hz, ablating from a stoichiometric YMnO_3_ target. Before thin-film deposition, each substrate was annealed in air at 1250 °C for 12 h. During the thin-film deposition, the substrate was kept at 750–800 °C in 0.12 mbar O_2_ environment. The thickness of the thin films was monitored using RHEED during growth and cross-checked with post-deposition X-ray reflectivity. The structural phase and orientation of the epitaxial films was determined using X-ray diffraction (Supplementary Fig. 1a, b). The X-ray characterization was performed using a PanAnalytical X’Pert^3^ MRD diffractometer. The thin-film topography (Supplementary Fig. 1c) was characterized using a Bruker Multimode 8 atomic force microscope.

### In situ SHG (ISHG)

The optical SHG signal was generated in reflection (see schematic in Supplementary Fig. 2a), in situ in the thin-film growth environment^32,33^. A pulsed Ti:Sapphire laser at 800 nm with a pulse duration of 45 fs and repetition rate of 1 kHz was converted using an optical parametric amplifier to a probe wavelength of 860 nm. This probe beam was incident on the sample with a pulse energy of 20 μJ on a spot size 250 μm in diameter. The generated light intensity (ISHG) was subsequently detected using a monochromator set to 430 nm and a photomultiplier system.

### Scanning transmission electron microscopy

Samples for STEM analysis were prepared by means of a FEI Helios NanoLab 600i focused-ion beam (FIB) operated at accelerating voltages of 30 and 5 kV. For the in situ heating experiments several thick lamellae of the 10-unit-cell YMnO_3_ film grown on YSZ were cut with the FIB and transferred to the Protochips Fusion Heating E-chips by using an EasyLift manipulator. After securing the FIB lamellae to the E-chips with Pt, the lamellae were further milled to electron transparency. The in situ heating studies were conducted using a Protochips Fusion 500 double-tilt holder with a Keithley 2636B power supply. A FEI Titan Themis with a probe CEOS DCOR spherical aberration corrector operated at 300 kV and equipped with a FEI CETA 2 camera was used for HAADF-STEM imaging and nanobeam electron diffraction pattern acquisition.

For the atomic-resolution HAADF-STEM data acquisition, a probe semi-convergence angle of 18 mrad was used in combination with an annular semi-detection range of the annular dark-field detector set to collect electrons scattered between 66 and 200 mrad. The temperature was raised from room temperature to 250 °C in steps of 25 °C at a rate of 1 °C s^−1^. At each step the temperature was kept constant for approximately 6–7 min in order to allow the sample to sufficiently stabilize (thermal drift occurs due to thermal expansion of the heating chip) and to acquire several time series consisting of 20 frames. Averages of the time series were subsequently obtained after both rigid and non-rigid registration using the Smart Align Software^40^.

### Density-functional calculations

For our DFT calculations of the ferroelectric slabs of YMnO_3_, we used the Vienna ab initio simulation package (VASP)^41–43^ which uses the projector augmented wave method^44^. We used the PBEsol + U^45^ functional and we consider 4s^2^4p^6^4d^2^5s^1^ for Y, 3s^2^3p^6^3d^6^4s^1^ for Mn and 2s^2^2p^4^ for O as valence. We used an artificial collinear up–up–down, down–down–up configuration for the magnetic moments on the Mn as is common in literature. To take into account electron correlations we used the Liechtenstein approach^46^ with a U of 4.5 eV and a J of 0.5 eV. In the relaxation process, we fixed the first unit cell and let the remaining atoms relax until the forces on the atoms were below 10^−3^ eV/Å. Our slab consists of 4 unit cells and around 50 Å of vacuum along the *c*-axis and we applied dipole corrections along the *c*-axis.

## Supplementary information

Supplementary Information

## Data Availability

The data that support the findings of this study are available from the corresponding authors upon request.
