# Supplementary material for: The ultrathin limit of improper ferroelectricity
Source: Nat Commun. 2019 Dec 6;10:5591. doi: 10.1038/s41467-019-13474-x (PMC6897979; doi:10.1038/s41467-019-13474-x)
Supplement: Supplementary file 1 — Supplementary Information [file 41467_2019_13474_MOESM1_ESM.pdf]

Supplementary Information for

The ultrathin limit of improper ferroelectricity

J. Nordlander *et al*.

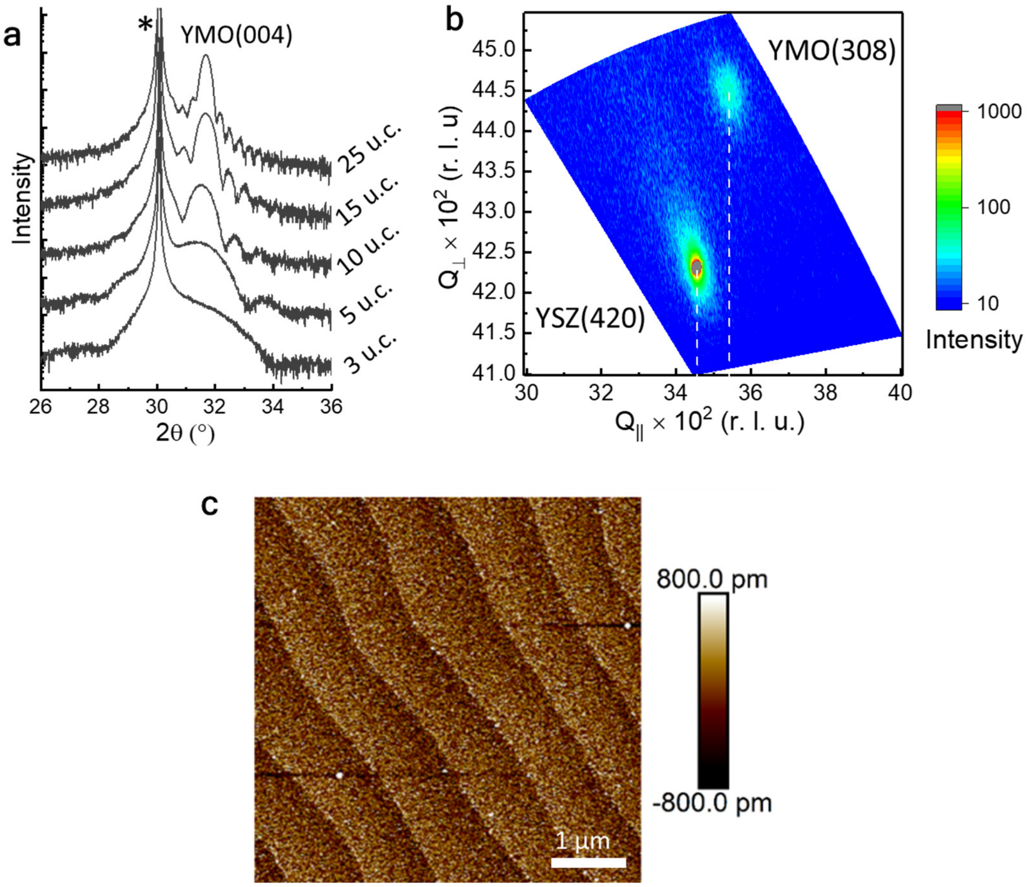

Supplementary Fig. 1. Structural characterization of the $YMnO_3$ thin films. a, θ-2θ scan in X-ray diffraction showing the substrate peak YSZ(111) (indicated by asterisk) and the *c*-axis oriented hexagonal film peak $YMnO_3$(004) for different film thicknesses. The thickness fringes around the film peak indicate the smoothness of the layer interfaces. The out-of-plane lattice parameter *c* of the films correspond to 11.3 Å. **b**, Reciprocal space mapping of the off-axis substrate and film peaks, YSZ(420) and $YMnO_3$(308), using X-ray diffraction for a 15 unit-cell film. The white dashed lines indicate the decoupled in-plane lattice parameters of the substrate and the film. **c**, Atomic force microscopy of the surface topography of a 15 unit-cell $YMnO_3$ thin film. The step terraces from the substrate surface are retained in the thin film topography.

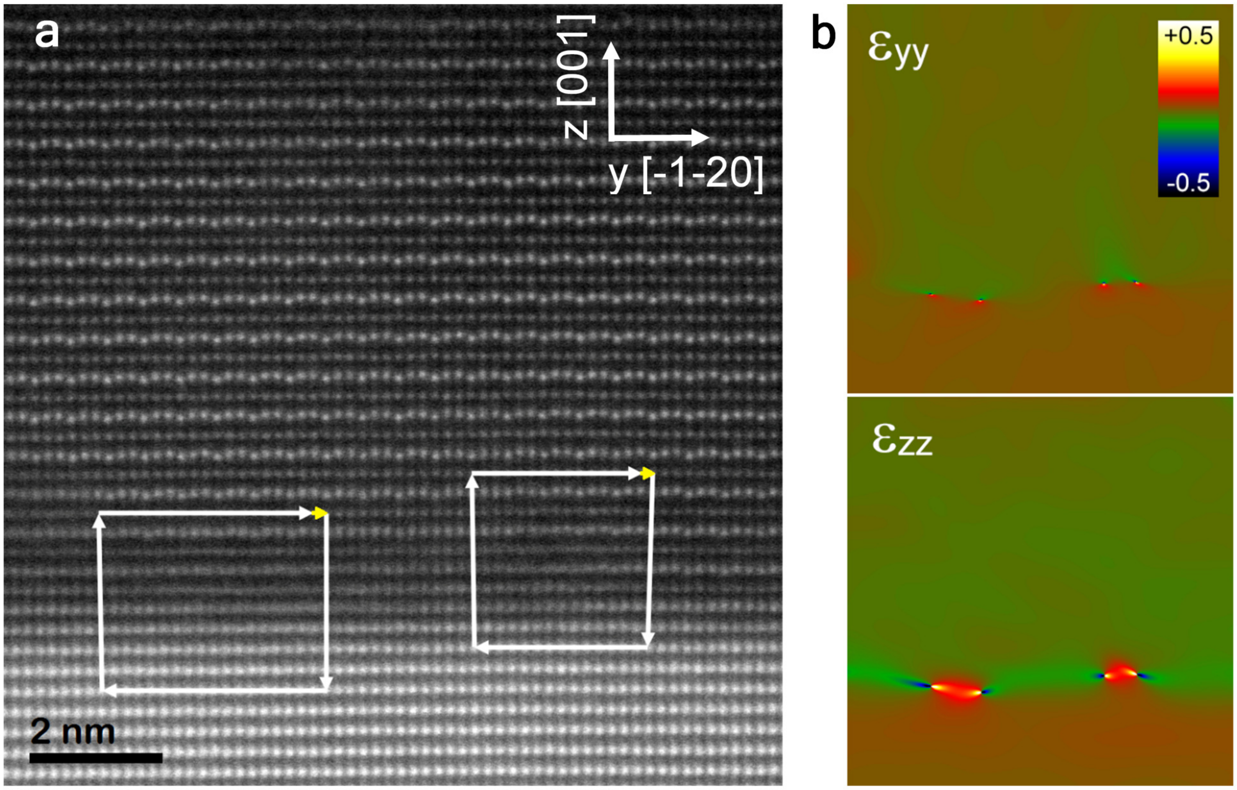

Supplementary Fig. 2. Strain mapping of a fully relaxed $YMnO_3$ thin film on YSZ(111). a, Representative HAADF-STEM image of the film-substrate interface in a 17 unit-cell film obtained as an average of a time series consisting of 15 frames acquired with 1 µs dwell time. Two Burgers circuits, built following the finish-start right-handed convention[10], are shown by the white arrows. Each Burgers circuit encloses a pair of partial dislocations and displays a global Burgers vector **b** = 1/3[-1-20] (shown by the yellow arrows), that is, an extra half-plane of atoms is inserted in the $YMnO_3$ film. **b,** Corresponding in-plane (||[-1-20]) and out-of-plane (||[001]) strain maps (ε_{yy} and ε_{zz}, respectively), obtained by geometric phase analysis[11], revealing the presence of two pairs of partial dislocations separated by ~5 nm. The color bar gives the strain change in percent. The ε_{yy} strain map shows retention of the $YMnO_3$ bulk-lattice parameters from the first unit cell mediated by misfit dislocations. The $YMnO_3$ film clearly shows a smaller in-plane lattice parameter (green color) than YSZ and thus does not adopt the larger lattice parameter from YSZ. In particular, the in-plane lattice parameter measured at the $YMnO_3$ film is -2.1(±0.6)% compared to YSZ, resulting in a lattice constant of 6.14 Å, in excellent agreement with a fully relaxed $YMnO_3$ film[12].

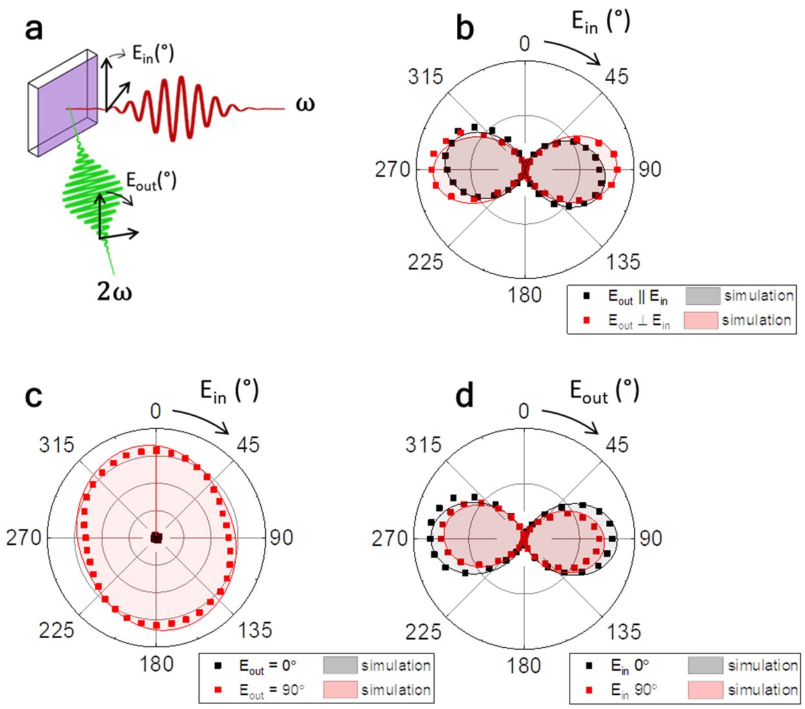

Supplementary Fig. 3. ISHG polarization dependence in the ferroelectric phase of $YMnO_3$ thin films. **a**, Schematic of the ISHG reflection geometry. The directions of incoming and detected light polarization (E_{in} and E_{out}) are given by their respective angles with respect to the vertical axis. **b-d**, Comparison between simulated and experimental SHG polarimetry confirms the polar 6*mm* symmetry associated to the ferroelectric phase in $YMnO_3$, where the amplitude of the SHG light is proportional to $\mathbf{P}_S$. The simulations fit to the I_{ISHG} when considering interference with a small background SHG contribution from the surface of the sample. The ISHG polarimetry is measured while: (**b**) E_{in} and E_{out} are rotated either in parallel (black data points) or orthogonal to each other (red data points), (**c**) E_{in} is rotated while E_{out} is fixed either at 0° (black data points) or 90° (red data points) and (**d**) E_{out} is rotated while E_{in} is fixed either at 0° (black data points) or 90° (red data points).

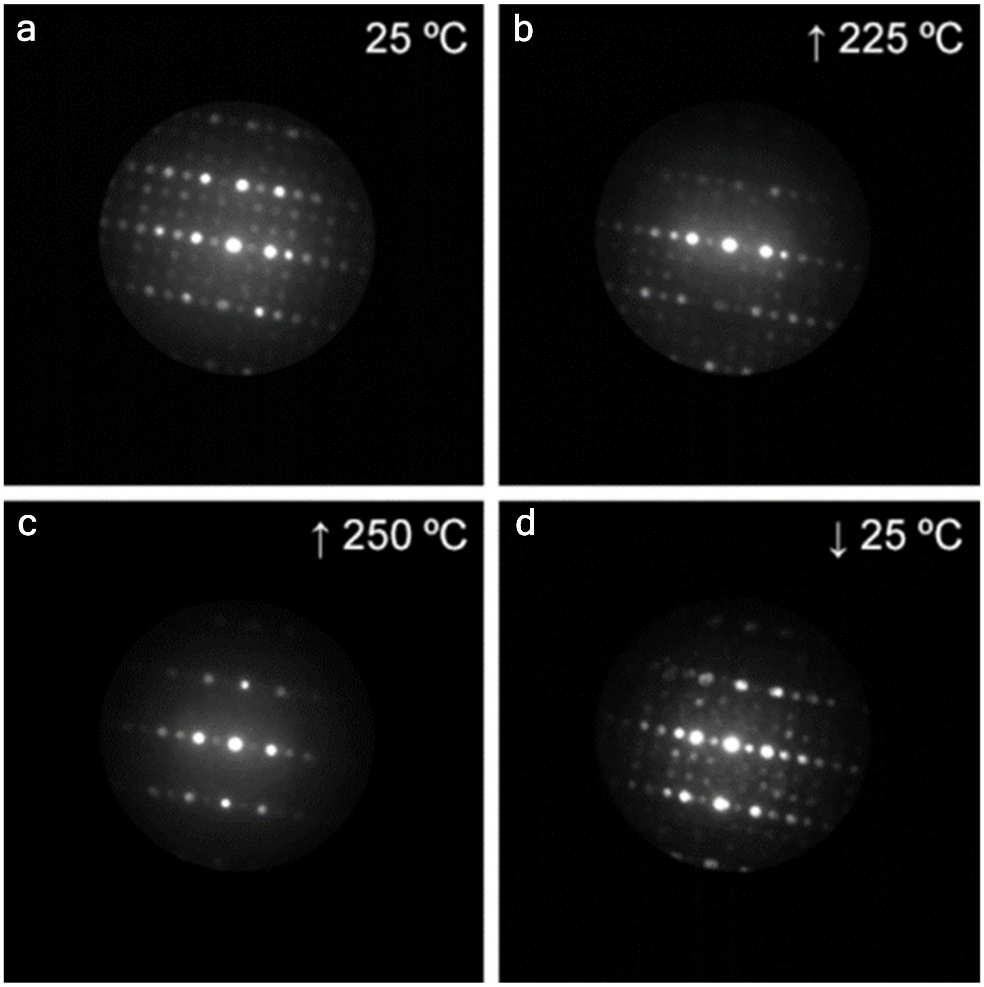

Supplementary Fig. 4. Temperature evolution of nanobeam electron diffraction patterns. Nanobeam electron diffraction of the 10 unit-cell $YMnO_3$ film showing that the characteristic superlattice reflections arising from the structural trimerization of the ferroelectric $YMnO_3$ lattice are visible at room temperature (**a**) and also weaker at 225 ºC (**b**) but vanish at 250 ºC (**c**). When cooling down the sample to 25 ºC (**d**) the superlattice reflections appear again demonstrating the reversible character of the structural phase transition. The sharp circular limitation of the diffraction pattern is given by the HAADF detector which is used to record STEM images needed to precisely position the electron probe on the area of interest.

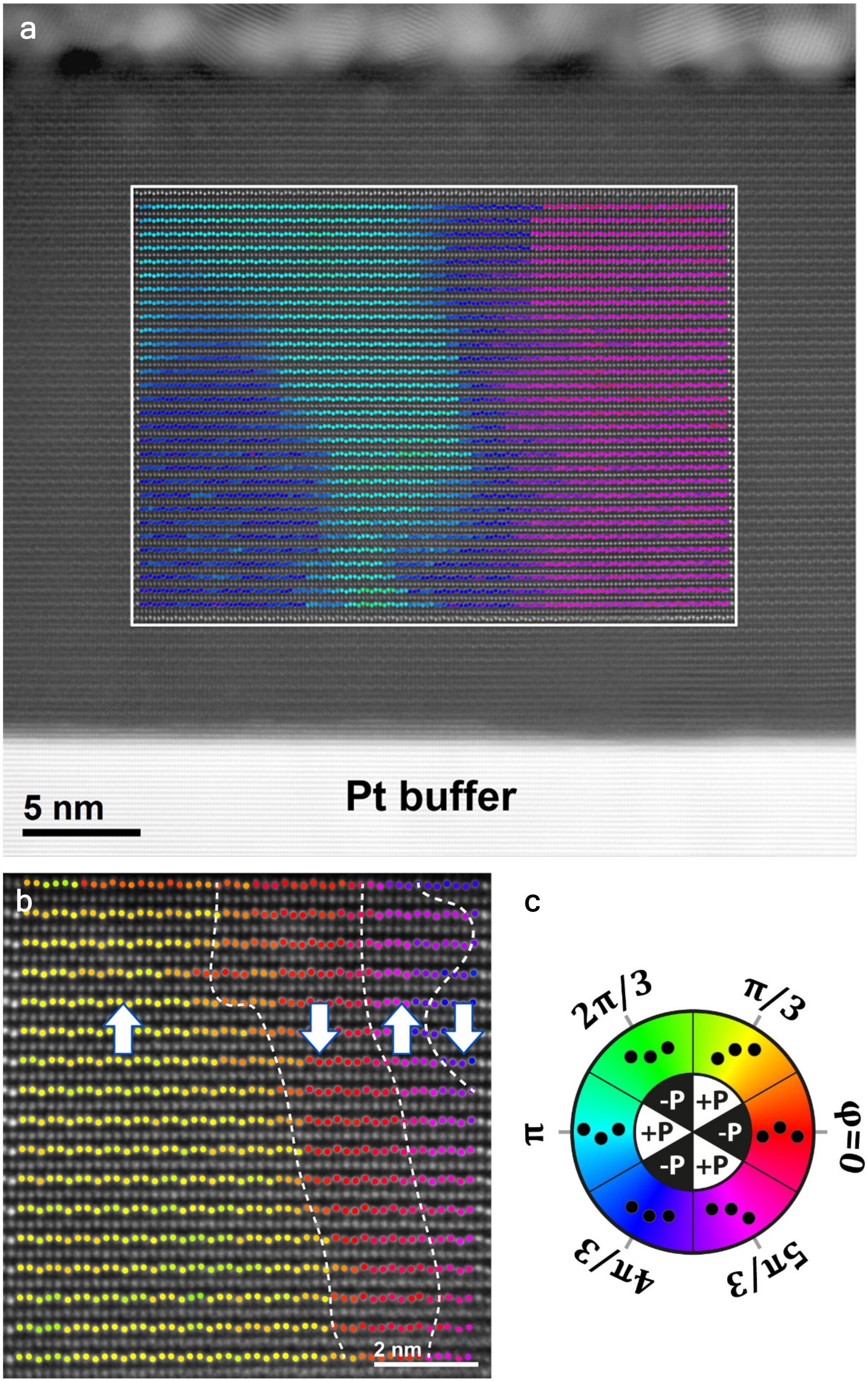
a
Pt buffer
5 nm
b
2 nm
c
2π/3
π/3
π
φ=0
4π/3
5π/3
-P
+P
+P
-P
-P
+P

Supplementary Fig. 5. Domain structure of a $YMnO_3$ film on charge-screening Pt(111)-buffered YSZ. **a**, Cross-sectional HAADF-STEM image displaying the 24 unit-cell thick $YMnO_3$ film, viewed along the [100] zone axis, and the bright-contrast epitaxial Pt buffer. The color overlay shows the crystal-lattice trimerization inside the white box. The domain structure of the $YMnO_3$ film is seen by mapping the trimerization angle φ as proposed by Holtz et al.[6]. The domains extend to a few nanometers in width and transform into each other according to the six allowed domain angles (φ = 0, π/3, 2π/3, π, 4π/3, 5π/3). **b**, HAADF-STEM image of another area displaying four domains and their corresponding domain walls indicated by the white dashed lines. The white arrows indicate the direction of the polarization $\mathbf{P}_s$. It is evident that the multidomain-state is present even as the depolarizing field is screened by a metallic Pt buffer-layer. **c**, Schematic showing the relation between the corrugation patterns of Y atoms for each of the six trimerization domain states, the corresponding tilt angles φ, direction of $\mathbf{P}_s$ and the associated color code.

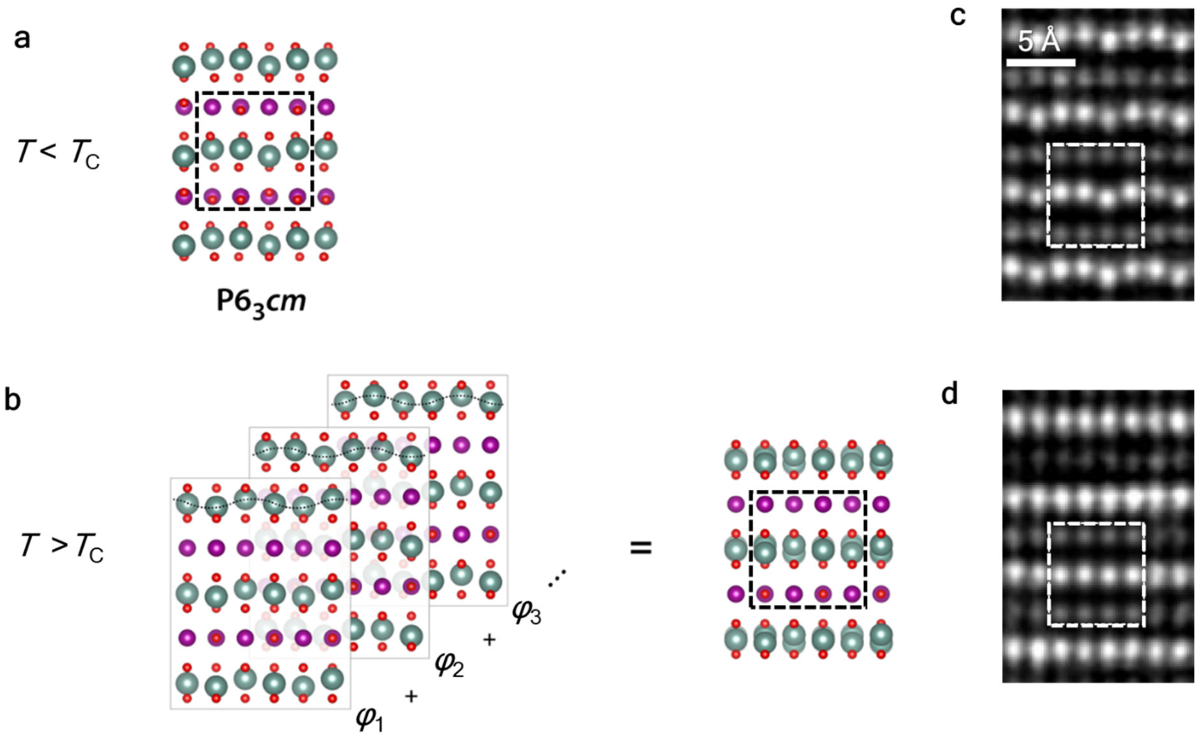

Supplementary Fig. 6. Multislice simulations of the temperature-dependent HAADF signal. The low temperature ($T < T_C$) phase is generated using the polar $P6_3cm$ structure (**a**), while the high-temperature ($T > T_C$) phase is generated as the superimposition of many states with different φ (continuum of states) (**b**). The simulated HAADF signals from (**a**) and (**b**) are based on the structure inside the black dashed boxes and shown as insets in white dashed boxes on top of the experimental data at T = 448 K < T_C (**c**) and T = 498 K ≥ T_C (**d**), respectively.

Supplementary Note 1: In-situ optical second harmonic generation (ISHG)

The amplitude of the I_{ISHG} signal depends on the incident and detected light polarization, which in the electric-dipole approximation for SHG can be expressed as[1]:

$$P_i(2\omega) = \varepsilon_0 \chi_{ijk} E_j(\omega) E_k(\omega). \tag{1}$$

The ferroelectric phase of $YMnO_3$ has a 6*mm* point-group symmetry with the following three independent tensor components of the second-order non-linear susceptibility:

$$\chi_{xxz} = \chi_{xzx} = \chi_{yyz} = \chi_{yzy}, \qquad \chi_{zxx} = \chi_{zyy}, \qquad \chi_{zzz},$$

where x, y, and z are the Cartesian coordinates of the hexagonal crystal lattice with $x \parallel a$ and $z \parallel c$. These tensor components become non-zero with the breaking of inversion symmetry due to a net spontaneous polarization and are thus proportional to the order parameter $\mathbf{P_s}$[2].

To confirm this polar signature of the SHG signal in the $YMnO_3$ thin films below the transition temperature, the light-polarization dependence (i.e. polarimetry) of the SHG signal was compared to the expected 6*mm* symmetry of the SHG obtained by simulations. The agreement seen between experimental data and simulation in Supplementary Fig. 3b-d was reached when taking the relative tensor components $\chi_{zxx} = 1, \chi_{xxz} = 0.16i, \chi_{zzz} = 0.3$ for the 6*mm* symmetry, and a small surface-induced SHG contribution was introduced in the simulation. Because of the high surface-to-volume ratio that is particular to thin films, SHG due to inversion-symmetry breaking at the surface often visibly interfere with the bulk-like SHG[3].

Based on the polarimetry in the polar phase of the $YMnO_3$ thin films, we chose a measurement geometry for the temperature-dependent SHG that singles out the dominating χ_{zyy}-related SHG component while minimizing the observed surface contribution. The samples were therefore oriented such that in tilted incidence (~ 45°), we project vertical light polarization (E_{in} = 0° in Supplementary Fig. 3) onto the vertical y-axis, and horizontal light polarization (E_{in} = 90° in Supplementary Fig. 3) onto the tilted ($x + z$) direction. We chose the polarization configuration E_{in} = 0° and E_{out} = 90° for the measurement of T_C^{film}. Hence, we could detect the intensity

$$I_{\mathrm{SHG}} \propto |P_z(2\omega)|^2 \propto |\chi_{zyy} E_y(\omega) E_y(\omega)|^2 \quad (2)$$

as a function of temperature. This signal was subsequently normalized by the square of the incident light intensity, I_{in}:

$$\frac{I_{\mathrm{SHG}}}{{I_{\mathrm{in}}}^2} \propto |\chi_{zyy}|^2 \propto |\mathbf{P}_s|^2, \quad (3)$$

to obtain the polarization dependence vs. temperature in the thin films.

Supplementary Note 2: Scanning transmission electron microscopy

Nanobeam electron diffraction patterns were acquired in the STEM mode by using the so-called microprobe mode (with the minicondenser lens excited) enabling a probe semi-convergence angle of 1 mrad. Representative nanobeam diffraction patterns acquired at room temperature, 225 ºC and 250 ºC are shown in Supplementary Fig. 4. At room temperature, the characteristic superlattice reflections arising from the structural trimerization of the ferroelectric $YMnO_3$ lattice are present. At 225 ºC, the intensity of the superlattice reflections start vanishing, and at 250 ºC only the main reflections due to the basic structure of the non-ferroelectric phase are visible. When cooling down the sample to room temperature the superlattice reflections appear again demonstrating the reversible character of the structural phase transition.

Fitting of the trimerization distortion from STEM images

The fitting of the atomic columns intensities was performed using an asymmetric Gaussian model[4,5]:

$$I(x,y) = I_0 + Z \exp\left[-\frac{1}{2(1-s^2)} \cdot \left(\left(\frac{x-x_0}{x_\mathrm{w}}\right)^2 + \left(\frac{y-y_0}{y_\mathrm{w}}\right)^2 - \left(\frac{2s(x-x_0)(y-y_0)}{x_\mathrm{w}y_w}\right)\right)\right], \quad (4)$$

where I_0 is the background, Z is the Gaussian amplitude, (x_0, y_0) the coordinates of the center of the peak, x_w and y_w the Gaussian variances and s the parameter describing the asymmetry of the peak.

The fitting of the trimerization associated with the K_3 mode was performed using the model proposed by Holtz et al.[6]:

$$u = Q\cos(\mathbf{q} \cdot \mathbf{u} - \varphi), \quad (5)$$

where $\mathbf{q}$ is the wave vector of the K_3 mode, $\mathbf{u}$ the position vector of the Y atom, Q the amplitude of the sinusoidal wave and φ its phase. The primary order parameter of the structural trimerization is hence given by $\mathbf{Q} = (Q \cos \varphi, Q \sin \varphi)$. Q is related to the maximum displacement ($1.5Q$) between

Y atoms and φ is associated to the six possible structural domains generated by the possible permutation of the ↑↑↓ and ↓↓↑ patterns.

The average |<**Q**>| values given in Fig. 3a and Fig. 4b were obtained by first calculating the average values of the **Q** vectors and then calculating their moduli. In Fig. 4b the |<**Q**>| values are shown for each temperature of the heating experiment with error bars given by the standard deviation of |<**Q**>| at $T > T_Q$ (where a homogeneous distribution is expected), thus disentangled from its thickness-dependent distribution at $T < T_Q$, (see Fig. 3a).

Calculation of the ellipticity of STEM images peaks

The expression given in Supplementary Eq. 4 resembles the equation of a two-dimensional elliptical Gaussian, given by:

$$f(x,y) = Z\exp[-(\alpha(x-x_0)^2 + \beta(x-x_0)(y-y_0) + \gamma(y-y_0)^2)]. \quad (6)$$

In order to extract quantitative information about the elliptic shape of the peak we need to retrieve the lengths of the ellipse semi-axes (a, b), that are related to the coefficients in Supplementary Eq. 6 by the relations:

$$\alpha = \frac{\cos^2\theta}{2a^2} + \frac{\sin^2\theta}{2b^2};\ \beta = \frac{\sin 2\theta}{2a^2} + \frac{\sin 2\theta}{2b^2};\ \gamma = \frac{\sin^2\theta}{2a^2} + \frac{\cos^2\theta}{2b^2}, \quad (7)$$

where θ is the rotation of the ellipse. A convenient way to retrieve the ellipse's parameters that does not require solving second order trigonometric equations (as would be the case using Supplementary Eqs. 4, 6 and 7) is to calculate these parameters starting from the explicit equation of the ellipse in Cartesian coordinates[7].

It is straightforward to demonstrate that the expression within the exponential function of Supplementary Eq. 4 can be rewritten in the implicit or explicit forms, given in Supplementary Eqs. 8 and 9 respectively:

$$A(x-x_0)^2+B(x-x_0)(y-y_0)+C(y-y_0)-a^2b^2=0, \tag{8}$$

$$Ax^2+Bxy+Cy^2+Dx+Ey+F=0, \tag{9}$$

where the parameters of the explicit form can be defined as:

$$A=\frac{1}{2(1-s^2)}\cdot\frac{1}{x_\mathrm{w}};\quad B=\frac{s}{(1-s^2)}\cdot\frac{1}{x_\mathrm{w}y_\mathrm{w}};\quad C=\frac{1}{2(1-s^2)}\cdot\frac{1}{y_\mathrm{w}};$$

$$D=-(By_0+2Ax_0);\quad E=-(Bx_0+2Cy_0);\quad F=-1. \tag{10}$$

It is worth to note that Supplementary Eq. 9 represents the equation of an ellipse if and only if $\Delta>0$ and $\delta>0$, where Δ and δ are defined as:

$$\Delta=4AC-B^2;\ \delta=CD^2+AE-BDE+F\Delta. \tag{11}$$

The ellipses semi-axes can be calculated using the formulas[7]:

$$a^2=\mu\frac{A+C+\sqrt{(A-C)^2+B^2}}{2};\ b^2=\mu\frac{A+C-\sqrt{(A-C)^2+B^2}}{2}, \tag{12}$$

where μ is defined as:

$$\mu=\frac{4\delta}{\Delta^2}\,. \tag{13}$$

The ellipticity (ε) is defined as the ratio between the major and minor semi-axes:

$$\varepsilon=\begin{cases}a/b, & a\geq b\,,\\ b/a, & a<b,\end{cases} \tag{14}$$

with values $\varepsilon \geq 1$ (where $\varepsilon = 1$ is the degenerate case for the spherical Gaussian).

HAADF-STEM Image simulations

The simulation of HAADF-STEM images was performed using a custom developed multislice frozen-phonon code[8], setting the beam convergence angle to 18 mrad and the collection angles for the HAADF detector in the range 70-190 mrad. The finite size of the source was set to 0.6 Å. The specimen thickness was set to 12.5 nm.

For the simulation of the low temperature ($T<T_C$) polar phase, we generated the slices starting from the $P6_3cm$ structure[9] (Supplementary Fig. 6a,c). In this case, the K_3 distortion produces the typical trimerization of Y atoms that is visible at all temperatures below T_C.

For the phase transformation to the paraelectric ($T>T_C$) phase, we assume an order-disorder model. In this case, the high-temperature phase corresponds to a continuum of states with different values of φ. In order to simulate this state of the system, we generated 20 different configurations with a random phase within the range $[0, 2\pi)$ and then averaged the simulated HAADF signals to obtain the continuum of states (Supplementary Fig. 6b). The superimposition of states with different φ is responsible for the appearance of elongated peaks in the HAADF signal, as described by the increase in the ellipticity for $T>T_C$. The experimental HAADF-STEM signals taken at 448 K and 498 K are shown in Supplementary Fig. 6c,d, superimposed with the simulated images (in the white boxes). The excellent comparison between simulated and experimental images supports an order-disorder mechanism for the phase transformation.

Supplementary references

1. Fiebig, M., Pavlov, V. V. & Pisarev, R. V. Second-harmonic generation as a tool for studying electronic and magnetic structures of crystals: review. *J. Opt. Soc. Am. B*. **22**, 96–118 (2005).

2. Sa, D., Valentí, R. & Gros, C. A generalized Ginzburg-Landau approach to second harmonic generation. *Eur. Phys. J. B - Condens. Matter Complex Syst.* **14**, 301–305 (2000).

3. Nordlander, J., De Luca, G., Strkalj, N., Fiebig, M. & Trassin, M. Probing ferroic states in oxide thin films using optical second harmonic generation. *Appl. Sci.* **8**, 570 (2018).

4. Yankovich, A. B. *et al.* Picometre-precision analysis of scanning transmission electron microscopy images of platinum nanocatalysts. *Nat. Commun.* **5**, 4155 (2014).

5. Campanini, M., Erni, R., Yang, C., Ramesh, R. & Rossell, M. D. Periodic Giant Polarization Gradients in Doped $BiFeO_3$ Thin Films. *Nano Lett.* **18**, 717–724 (2018).

6. Holtz, M. E. *et al.* Topological defects in hexagonal manganites: Inner structure and emergent electrostatics. *Nano Lett.* **17**, 5883–5890 (2017).

7. Horwitz, A. https://arxiv.org/abs/1705.09845 (2017).

8. Erni, R., Heinrich, H. & Kostorz, G. Quantitative characterisation of chemical inhomogeneities in Al–Ag using high-resolution Z-contrast STEM. *Ultramicroscopy*. **94**, 125–133 (2003).

9. Skjærvø, S. H. *et al.* Unconventional Continuous Structural Disorder at the Order-Disorder Phase Transition in the Hexagonal Manganites. *Phys. Rev. X.* **9**, 031001 (2019).

10. Hirth J. P. & Lothe J. Theory of Dislocations, 2nd ed. (Wiley, New York, 1982).

11. Hÿtch, M. J., Snoeck, E. & Kilaas, R. Quantitative measurement of displacement and strain fields from HREM micrographs. *Ultramicroscopy* **74**, 131–146 (1998).

12. Katsufuji, T. *et al.* Crystal structure and magnetic properties of hexagonal $RMnO_3$ (R= Y, Lu, and Sc) and the effect of doping. *Phys. Rev. B* **66**, 134434 (2002).
